# LeGO-LOAM-SC: An Improved Simultaneous Localization and Mapping Method Fusing LeGO-LOAM and Scan Context for Underground Coalmine

**DOI:** 10.3390/s22020520

**Published:** 2022-01-11

**Authors:** Guanghui Xue, Jinbo Wei, Ruixue Li, Jian Cheng

**Affiliations:** 1 School of Mechanical Electronic and Information Engineering, China University of Mining and Technology, Beijing 100083, China; xgh@cumtb.edu.cn (G.X.); vjinbo@163.com (J.W.); lrx18811043945@163.com (R.L.); 2 Key Laboratory of Intelligent Mining and Robotics, Ministry of Emergency Management, Beijing 100083, China; 3 Research Institute of Mine Big Data, China Coal Research Institute, Beijing 100013, China

**Keywords:** simultaneous localization and mapping, LeGO-LOAM, scan context, loop detection, ICP graph optimization, unmanned vehicle

## Abstract

Simultaneous localization and mapping (SLAM) is one of the key technologies for coal mine underground operation vehicles to build complex environment maps and positioning and to realize unmanned and autonomous operation. Many domestic and foreign scholars have studied many SLAM algorithms, but the mapping accuracy and real-time performance still need to be further improved. This paper presents a SLAM algorithm integrating scan context and Light weight and Ground-Optimized LiDAR Odometry and Mapping (LeGO-LOAM), LeGO-LOAM-SC. The algorithm uses the global descriptor extracted by scan context for loop detection, adds pose constraints to Georgia Tech Smoothing and Mapping (GTSAM) by Iterative Closest Points (ICP) for graph optimization, and constructs point cloud map and an output estimated pose of the mobile vehicle. The test with KITTI dataset 00 sequence data and the actual test in 2-storey underground parking lots are carried out. The results show that the proposed improved algorithm makes up for the drift of the point cloud map, has a higher mapping accuracy, a better real-time performance, a lower resource occupancy, a higher coincidence between trajectory estimation and real trajectory, smoother loop, and 6% reduction in CPU occupancy, the mean square errors of absolute trajectory error (ATE) and relative pose error (RPE) are reduced by 55.7% and 50.3% respectively; the translation and rotation accuracy are improved by about 5%, and the time consumption is reduced by 2~4%. Accurate map construction and low drift pose estimation can be performed.

## 1. Introduction

As an important traditional energy industry in China, the coal industry is an important part of China’s national economy. Intelligent coal mines are the core technical support for the high-quality development of the coal industry, which is of great significance in improving the safety level of coal mine production and in ensuring the stable coal supply. SLAM is one of the ways to build the underground environmental map of complex coal mines and its own intelligent positioning for coal mine operation vehicles. It is one of the key technologies to realize unmanned driving and autonomous operation in a coal mine [1].

Scholars at home and abroad have carried out a large number of studies on SLAM algorithms based on vision [2,3,4,5] and LiDAR [6,7,8,9,10]. Due to advantages of intuitive mapping, high ranging accuracy, easily unaffected by the variation of illumination and view angle, and its ability to operate in all weather conditions [11], Lidar is widely used in the field of unmanned driving [12,13,14,15] and is more suitable for SLAM in Complex and changeable coal mine environments with poor light conditions. Huber and Vandapel [16] used a high-precision laser scanner to build a high-precision three-dimensional geological model of a coal mine, but this method needs post-processing of surveying data, which cannot meet the requirements of real-time mapping and positioning of the coal mine environment, and the cost is high. Ren Z. et al. [17] studied the lightweight loop detection and optimization algorithm based on rules and Generalized ICP (GICP) [18], and proposed the SLAM optimization method based on GICP 3D point cloud registration, but the positioning and mapping accuracy still needs to be improved. Considering the real-time and accuracy of positioning and mapping, there are still many problems to be solved in coal mine underground environment SLAM.

LiDAR Odometry and Mapping (LOAM) [19,20] is presently the most representative real-time 3D laser SLAM algorithm based on feature matching. It has a small amount of calculation and motion compensation, but there is no loop detection, resulting in drift error accumulating over time and poor mapping accuracy, and degradation problems in an open environment. In view of the lack of LOAM algorithm, Shan T [21] added loop detection to find loop points by combining ICP and Euclidean distance, and carried out lightweight and ground optimization processing in feature extraction, and proposed Light weight and Ground-Optimized LiDAR Odometry and Mapping (LeGO-LOAM) to achieve a similar or better accuracy under the condition of reduced computing resources. However, sometimes loop detection has errors or identification omissions. HDL-Graph-SLAM [22] integrates the optimized Normal Distributions Transform (NDT) [23] point cloud registration algorithm, and uses the method of accessing historical key frames to perform loop detection and matching on the scene scanned by the point cloud of the current frame. It has good stability, but the mapping speed is slow and the point cloud matching accuracy is not high. IMLS-SLAM [24] proposed a scan-to-model matching framework based on implicit moving least squares (IMLS) surfaces, which can provide accurate attitude estimation, but it is not real-time estimation. SuMa [25] is a motion estimation and mapping method based on surfel map. It uses projection data association to estimate the dense registration of each projection point. It is more robust to missing features or missing data and can establish a global consistent map in a large-scale environment only based on laser point cloud data with a high accuracy, but the efficiency is limited. Reference [26] proposed an optimization method of loop detection based on ground plane constraints and segmatch to realize low drift positioning and the construction of dense 3D point cloud images. Loop detection methods based on fast point feature histogram (FPFH) [27] and Gasalt3D [28] local descriptors require key point extraction and large-scale local geometric calculation, and has low loop detection efficiency. Rizzini D L [29] studied the global descriptor-based GLAROT loop detection method, but its efficiency is still low. Giseop Kim and ayoung Kim of KAIST University in Korea proposed a scan context loopback detection algorithm [30], which uses a non-histogram global descriptor to realize fast and effective search and matching of current and historical frame data. It has the characteristics of high precision, and low time-consumption and computational costs. It is an efficient and robust loopback detection method.

In view of the real-time and accuracy requirements of map construction of the coal mine underground environment, and considering the high-precision, low-cost, efficient and robust characteristics of the scan context, this paper uses a scan context algorithm to optimize the LeGO-LOAM loopback detection module, and uses an ICP algorithm to optimize the global map obtained by loop, and proposed a LeGO-LOAM-SC SLAM algorithm fusing Scan Context and LeGO-LOAM to improve the accuracy, real-time and robustness of coal mine underground map construction, and also evaluated the performance of the proposed algorithm with the KITTI data set 00 sequence data and the point cloud data collected experimentally in an underground simulation scene, so as to explore a better SLAM algorithm and to provide technical support for map construction and unmanned driving of the coal mine underground environment.

## 2. Algorithm Principle and Improvement

### 2.1. LeGO-LOAM

**Figure 1:**
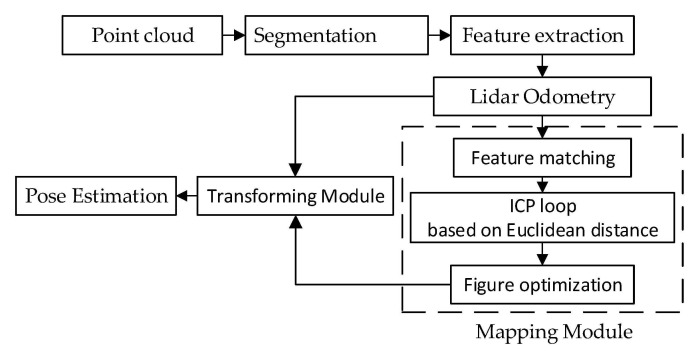
System structure diagram of the LeGO-LOAM algorithm.

LeGO-LOAM is a lightweight real-time positioning and mapping algorithm based on 3D LiDAR, proposed by Shan T et al. on the basis of an LOAM algorithm in 2018. It is mainly composed of point cloud segmentation, feature extraction, LiDAR measurement, LiDAR mapping and transformation fusion, as shown in Figure 1.

Let P_t_ = {p_1_, p_2_, …, p_n_} be the point cloud data obtained by LiDAR at time t, where p_i_ is a point in P_t_ and its Euclidean distance to LiDAR is indicated by r_i_.

To improve the processing efficiency and the accuracy of feature extraction, through the point cloud segmentation module, the point cloud P_t_ is divided into different clusters and is marked as ground points or segmentation points. At the same time, three features of each point, namely, the label of the point, the row and column index in the depth map and the distance value, are obtained for subsequent modules. First, the point cloud is projected onto a depth map, and the point P_i_ in P_t_ is mapped to a pixel on the depth map. Before segmentation, the ground plane of the depth map is estimated to extract the ground features, and the points representing the ground plane are marked as ground points, which do not participate in point cloud segmentation. Then, the point cloud is divided into many clusters by an image segmentation method, and the points in the same cluster are marked as an exclusive label. The ground points are kind of exclusive clusters. When using segmented point cloud for fast and reliable feature extraction, clusters with fewer than 30 points are ignored to reduce the insignificant or unreliable features formed by small objects such as leaves in a noisy environment.

The feature extraction module extracts edge and planar features from ground points or segmentation points. The extraction process is as follows:

(1) Let S be the set of continuous points in the same row in the depth map and calculate the roughness c of the point p_i_
  (1) $$c=\frac{1}{\left|S\right|\cdot \left\|r_{i}\right\|}{\left\|\sum_{{}_{j\in S,j\neq i}}^{}\left(r_{j}-r_{i}\right)\right\|}_{\;}$$
  where, r_i_ and r_j_ are the Euclidean distances from points p_i_ and p_j_ in set S to LiDAR, respectively.
(2) Divide the depth map horizontally into several equal sub images to extract features evenly.
(3) Segment different types of features according to the set threshold c_th_. The points with roughness value c greater than c_th_ are segmented into edge feature points, and the points less than c_th_ are segmented into plane feature points. The non-ground edge feature point n***F***_e_ with the largest roughness c and the plane feature point n***F***_p_ with the smallest roughness c in the ground or segmentation points are selected from each row of the sub image to obtain the edge feature point set ***F***_e_ and the plane feature point set ***F***_p_ in all sub images. Then, the non-ground edge feature n***F***_e_ with the largest roughness c and the ground plane feature n***F***_p_ with the smallest roughness c are selected from each row of the sub image to obtain the edge feature set *F*e and the plane feature set *F*_p_ in all sub images. Obviously, *F*_e_ ⸦ ***F***_e_, *F*_p_ ⸦ ***F***_p_.

The LiDAR odometry module estimates the motion of the robot in two consecutive frames, and uses the features extracted from the feature extraction module to find the correlation transformation of the robot position in the continuous scanning frame. During the estimation process, the label matching is used to narrow the matching range and to improve the accuracy, and the two-step Levenberg-Marquardt (L-M) optimization method is used to find the conversion relationship between two consecutive frames. The first step is to use the ground plane features *F*p to obtain [t_z_, θ_roll_, θ_pitch_]; the second step is to match the edge features extracted from the segmented point cloud to obtain the transformation [t_x_, t_y_, θ_yaw_], and then the 6-dimensional transformation between two consecutive scans is obtained finally by the fusion [t_z_, θ_roll_, θ_pitch_] and [t_x_, t_y_, θ_yaw_], which reduces the computational time by approximately 35% with a similar accuracy.

The LiDAR mapping module matches the features in the feature set {**F**_e_^t^, **F**_p_^t^} with the surrounding point cloud map Q^−t−1^ to further refine the attitude transformation, then uses the final transformed pose obtained by L-M optimization to add the spatial constraints between the new node of the point cloud map and the historically selected node, and adds new constraints through loop detection, then sends the pose map to GTSAM for graph optimization and updates the estimated pose by sensor. The transforming module fuses the pose estimation results from the LiDAR odometry module and the LiDAR mapping module, and outputs the final pose estimation.

The loop detection module of LeGO-LOAM uses a k-dimensional tree (KD tree) model to find the historical pose similar to the current pose and its nearby point clouds based on Euclidean distance, uses ICP to calculate its matching degree and estimates the pose, and uses the robot pose of the most similar historical frame to constrain the current robot pose estimation and updates the point cloud map to obtain the global consistency map. The loop algorithm has a large amount of calculation and a low detection efficiency. To give consideration to real-time and accuracy, a lower frequency loop detection is adopted, and there is still a large cumulative error in the mapping of long-range and large scenes.

### 2.2. Scan Context

The scan context algorithm, proposed by Giseop Kim and Ayoung Kim of KAIST University, uses global descriptors of non-histograms to enable a faster and efficient search of “context” (current/previous data). Scan context transforms 3D point clouds into 2.5D by dimensionality reduction, and uses a search algorithm to match the point cloud data of the current frame and the historical frame to realize loopback detection.

**Figure 2:**
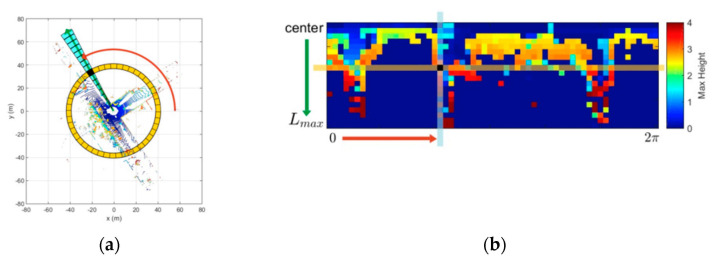
Construction diagram of scan context global descriptor. (**a**) Top view of one frame of point cloud; (**b**) Rectangular image expanded from the top view.

Figure 2 shows the structure diagram of scan context global descriptor. For a frame of point cloud data scanned by LiDAR, the top view is obtained from the top of 3D point cloud (as shown in Figure 2a), and the polar coordinate system is established with the LiDAR position as its origin. Twenty rings are divided outward from origin, and each ring is divided into 60 equal parts, namely 1200 grids, taking the maximum height (Z value) of the points in each grid as the grid value. Then the top view is expanded radially into 20 rows and 60 columns of rectangular images (Figure 2b), the average values of each row and each column are calculated respectively, and the two vectors—ring key and sector are obtained as global descriptors.

**Figure 3:**
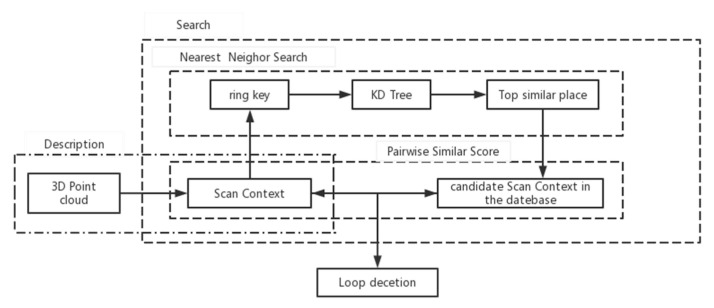
Loop detection algorithm overview.

The flow of scan context loopback detection algorithm is shown in Figure 3. The rectangular image scan context is constructed by using the point cloud data scanned at one time, the KD tree is constructed by using the ring key vector, the nearest neighbor search is performed, multiple similar frames that may loopback with the current frame and their ring key translation values are found, the similarity score is calculated, and the similar frames with high scores are screened out; then, the minimum offset and the similarity score is calculated sector by sector, the frame with the highest similarity score is selected as the loopback frame, and the pose relationship between the current frame and the loopback frame is solved, and the loop detection is realized.

### 2.3. Improved Algorithm Principle

**Figure 4:**
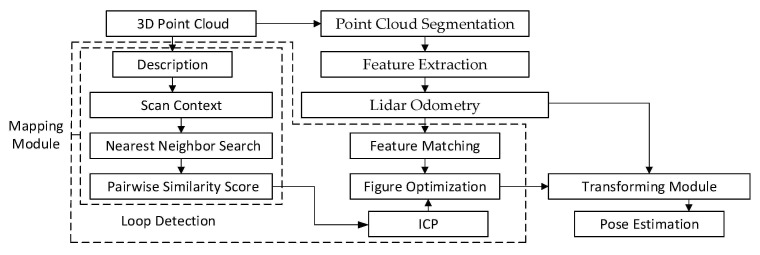
System structure diagram of the LeGO-LOAM-SC algorithm.

To overcome the shortcomings of the LeGO-LOAM loop detection algorithm, the scan context algorithm is used to replace the ICP loop detection method based on the Euclidean distance in the LeGO-LOAM algorithm, and the pose constraints are calculated by ICP and added to GTSAM for global pose optimization to build a global map, named as the LeGO-LOAM-SC algorithm. This algorithm reduces the dimension of the point cloud by integrating the scan context loopback algorithm, with a small amount of computation and a fast loopback detection, to improve the mapping accuracy and efficiency. The algorithm block diagram is shown in Figure 4.

The LeGO-LOAM-SC algorithm flow is as follows:

(1) Read the point cloud data collected by LiDAR, divide each frame of point cloud Pt by the point cloud segmentation module into different clusters and mark them as ground points or segmentation points. Meanwhile, three characteristics of each point, namely, the label of the point, the row and column index in the depth map and the distance value, are obtained and the point cloud that cannot be clustered is removed.
(2) By the feature extraction module, calculate the feature point roughness *c*, and extract the edge feature points n*F*_e_ and the plane feature points n*F*_p_ according to the roughness c ranking.
(3) The LiDAR odometry module uses edge feature points and plane feature points to obtain the pose transformation matrix through a two-step L-M optimization, and obtains the spatial constraints between two continuous frame point clouds.
(4) The LiDAR mapping module matches the features in {***F***_e_^t^, ***F***_p_^t^} with the surrounding point cloud Q^−t−1^ to further refine the posture transformation, and then uses the final transformed pose obtained by L-M optimization to add the spatial constraints between the new node of the point cloud map and the historically selected node, sends the pose map to GTSAM for map optimization, updates the sensor estimated attitude and updates the current map.
(5) Further eliminate the drift of point cloud map through a scan context loopback detection algorithm. The process is as follows:
  (a) Encode point cloud data
    The 3D point cloud is divided into *N_s_* axial sector and *N_r_* radial ring bin in LiDAR coordinates at equal intervals, as shown in Figure 2a. If the maximum sensing range of the LiDAR is *L*_max_, the radial gap between the rings is *L*_max_/*N_r_*, and the central angle of the sector is equal to 2π/*N_s_*. Generally, *N*_s_ and *N*_r_ are set at 60 and 20, respectively.
    Set ***P***_ij_ be a point set that belongs to the overlapping bins of the i_th_ ring and j_th_ sector, and take the maximum height of the point cloud p in the point set ***P***_ij_ as the value of the the radial ring bin, then the bin coding function is:
    (2) $$\phi \;\left(P_{\mathrm{ij}}\right)=\underset{P\in P_{\mathrm{ij}}}{\max}z(p)$$
    where *z*( ) is a function of the z-coordinate value of point cloud *p*, and the empty bin is assigned a zero value. Then the scan context *I* is finally expressed as the *N_r_* × *N_s_* matrix, as follows:
    (3) $$I=\left(a_{\mathrm{ij}}\right)\in R^{Nr\times Ns}{,\;a}_{\mathrm{ij}}=\phi \;\left(P_{\mathrm{ij}}\right)$$
  (b) Generate a scan context
    The point cloud and candidate point cloud to be queried are retrieved, the distance between two column vectors at $c_{j}^{q}$ and $c_{j}^{c}$ of the same index by cosine distance is calculate, and the distance function is normalized by dividing the sum of the distances between columns in the same index by the total number of columns *N_s_*.
    (4) $$d\left(I^{q},{Ι}^{c}\right)=\frac{1}{N_{s}}\overset{N_{s}}{\underset{j=1}{\sum }}(1-\frac{c_{j}^{q}\cdot c_{j}^{c}}{\left\|c_{j}^{q}\|\|c_{j}^{c}\right\|})$$
    where, *I^q^* and *I^c^* are the scan context obtained from the point cloud to be queried and the candidate point cloud, respectively.
    Get the vector K. The displacement of LiDAR sensor coordinates relative to the global coordinates will change the column order. Use all possible column displacement scanning contexts to calculate the distance and find the minimum distance. Set $I^{c}$ shift *n* columns to get matrix $I_{n}^{c}$, then the column movement number *n* and the corresponding distance of the best alignment can be obtained according to the minimum distance,
    (5) $$D\left(I^{q},I^{c}\right)=\underset{n\in \left[N_{s}\right]}{min}d\left(I^{q},I_{n}^{c}\right)$$
    (6) $$n^{*}=\underset{n\in \left[N_{s}\right]}{argmin}d\left(I^{q},I_{n}^{c}\right)$$
    Each row *r* of scan context is encoded into a real value by the ring coding function *ψ*, and the ring key is represented by the *N_r_* dimensional vector *K*, whose element is taken from the nearest ring to the farthest ring from the LiDAR.
    (7) $$k=\left(\psi \left(r_{1}\right),\cdots ,\psi \left(r_{N_{r}}\right)\right),\;\;\psi :r_{i}\to R$$
  (c) Confirm the index of the loopback frame.
    Vector *K* is used to construct the key of KD tree. The queried ring key is used to find similar ring keys and their corresponding scan indexes. Use distance $D(I^{q},I^{c})$ to compare the candidate scan context with the scan context to be queried,
    (8) $$c^{*}=\underset{c_{k}\in C}{arcmin}D\left(I^{q},I^{c_{k}}\right),\begin{array}{cc} s.t & D<\tau \end{array}$$
    where *c* is a set of candidate indexes extracted from KD tree, *τ* is the given threshold, and *c*^∗^ is the index where it is determined to be looped.
(6) Combine *m* keyframes near *c^∗^* into a local map, convert the current key frame to the world coordinate system, register with the local map and calculate the registration score by the ICP method. If the registration score is less than the given threshold, the loopback is considered successful, and the pose constraints between the loopback frame and the current frame is obtained. The constraints are added to GTSAM for map optimization and to update the point cloud map. The transform fusion module fuses the position and position estimation results from the LiDAR range meter module and the LiDAR mapping module, and outputs the final position and the position estimation.

## 3. The KITTI Dataset Test

The test software environment is Ubuntu 18.04, ROS melodic, PCL 1.10, GTSAM 4.0.3, Python 2.7.17, and the hardware configuration is 8 GB of RAM, Intel Core i3-4100M, and NVIDIA GeForce 940M. The test data set adopts KITTI data set 00 sequence.

### 3.1. Mapping Effect

**Figure 5:**
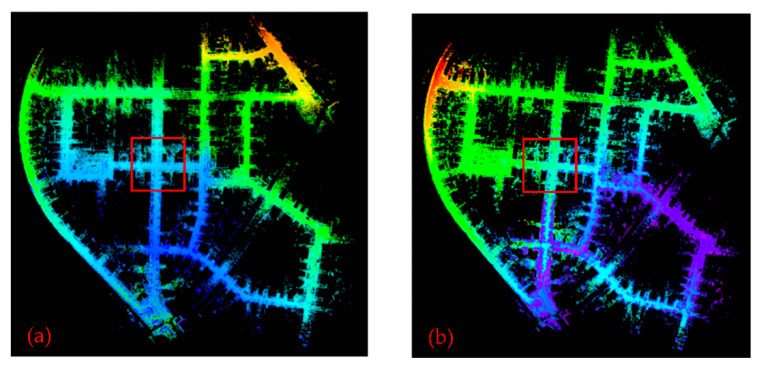
Point cloud map constructed by two algorithms using KITTI dataset 00 sequence point cloud data. (**a**) By LeGO-LOAM; (**b**) by Lego-Loam-SC.

**Figure 6:**
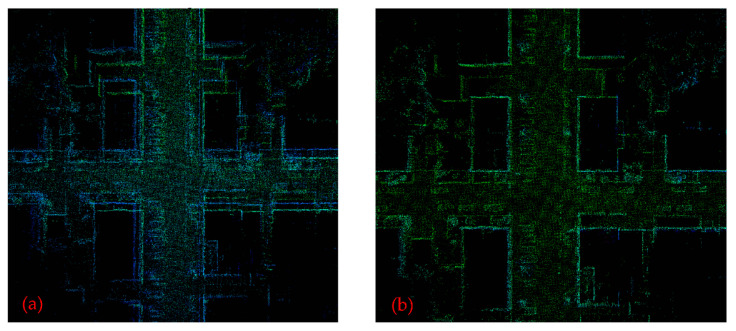
Partial enlarged view of the red box part of Figure 5. (**a**) By LeGO-LOAM; (**b**) by LeGO-LOAM-SC.

Figure 5 shows a point cloud map of the 00 sequence of the KITTI dataset constructed by LeGO-LOAM and LeGO-LOAM-SC, and the red box is where the scanning start point and end point are located. Figure 6 is a partial enlarged view of the red box part of Figure 5. It can be seen that when mapping by LeGO-LOAM algorithm, the loopback effect is poor and there is a phenomenon of point cloud map drift, and the initial map and loopback map do not coincide. While mapping by LeGO-LOAM-SC, the initial map and loopback map show good consistency, and the phenomenon of point cloud map drift make up.

### 3.2. Track Comparison

**Figure 7:**
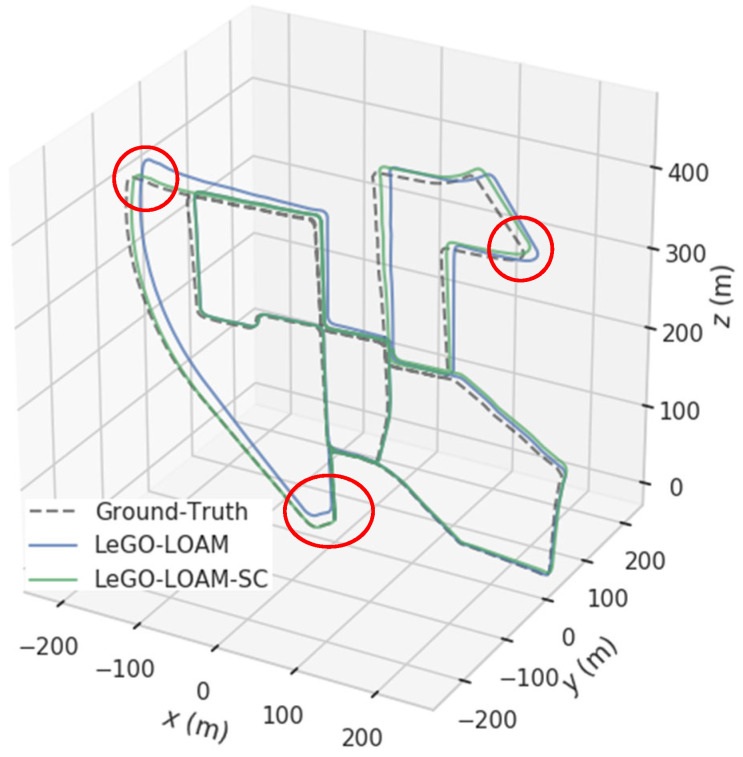
Comparison of tracks diagram obtained by LeGO-LOAM-SC and LeGO-LOAM.

Evo is a Python package for the evaluation of odometry and SLAM, and provides executables and a small library for handling, evaluating and comparing the trajectory output of odometry and SLAM algorithms [31,32]. It supports many trajectory formats: ‘TUM’ trajectory files, ‘KITTI’ pose files, ‘EuRoC MAV’ (.csv groundtruth and TUM trajectory file), ROS and ROS2 bagfile, etc. evo has several advantages over other public benchmarking tools: common tools for different formats; algorithmic options for association, alignment, scale adjustment for monocular SLAM etc.; flexible options for output, plotting or export (e.g., LaTeX plots or Excel tables); a powerful, configurable CLI that can cover many use cases; modular core and tools libraries for custom extensions; faster than other established Python-based tools. The motion tracks were extracted using the evo tool, as shown in Figure 7. As can be seen from the figure that the motion trace generated by LeGO-LOAM-SC had more coincidence with the real trace. At a sharp turning angle (circled in red in the Figure 7), LeGO-LOAM failed to smooth the loop, while the loop of LeGO-LOAM-SC algorithm is smoother and the effect is better.

### 3.3. Estimate Trajectory Length Deviation and Time

The evo tool is used to analyze the track length and its deviation, CPU occupancy and time consumption obtained by LeGO-LOAM and LeGO-LOAM-SC, as shown in Table 1. It can be seen that compared with the LeGO-LOAM algorithm, the LeGO-LOAM-SC optimizes the loop detection link and the actual track length is closer to the real track length, the deviation is reduced by 47.8%, the time consumption is reduced by 2%, and the CPU occupancy is reduced by 6%, indicating that the improved algorithm has a higher mapping accuracy, a better real-time performance and a lower resource occupancy.

### 3.4. Absolute Trajectory Error and Relative Pose Error

Table 2 shows the maximum error, minimum error and mean square error of ATE and RPE. It can be seen that the maximum error, minimum error and mean square error of ATE by LeGO-LOAM-SC algorithm are reduced by 49.4%, 79.1% and 55.7% respectively, and that the maximum error, minimum error and mean square error of RPE are reduced by 62.9%, 25.0% and 50.3% respectively, which shows that the LeGO-LOAM-SC algorithm has a better accuracy in long-distance scenes, and is suitable for the mapping of large-scale scenic cloud maps with a higher accuracy.

## 4. Experimental Verification

**Figure 8:**
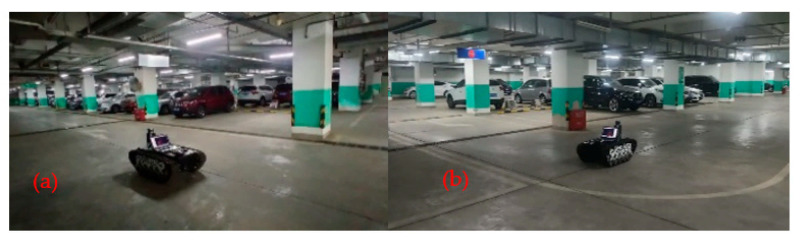
Experimental environment. (**a**) Scene #1 of Basement 1 parking lot; (**b**) Scene #2 of Basement 2 parking lot.

A tracked car equipped with 16-line lidar is used to simulate the mobile operation vehicle in a coal mine. The equipped LiDAR is RS-LiDAR-16, with a measurement range of 150 m, an accuracy of ±2 cm, a vertical perspective of 30°, a horizontal perspective of 360°, a vertical angle resolution of 2°, a horizontal angular resolution of 0.2°, and a rotation rate of 10 Hz. The testing was carried out at a large underground parking lot with two floors, as shown in Figure 8. The parking lot has a large scene and rich environmental characteristics. There are bumpy roads such as deceleration belts and sewers, which can test the positioning and mapping accuracy and robustness of the algorithm.

**Figure 9:**
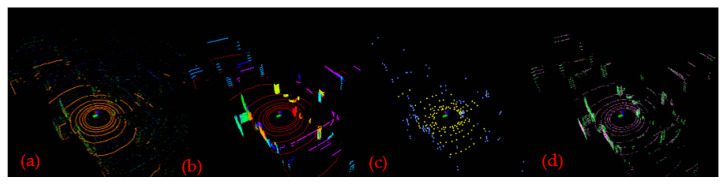
The results of feature extraction at every stage. (**a**) The original point cloud collected by LiDAR; (**b**) the ground points (in red) and the segmentation points (in others) after point cloud segmentation; (**c**) the edge feature points set n***F***_e_ (in blue) and the plane feature points set n***F***_p_ (in orange); (**d**) the edge feature *F*_e_ (in green) and the plane feature *F*_p_ (in pink).

Feature extraction is the key module of point cloud map construction. For a frame of point cloud data obtained by RS-LiDAR-16 LiDAR, the feature extraction was performed using LeGO-LOAM-SC, and the results of feature extraction at every stage were shown in Figure 9. 

In Figure 9, (a) shows the original point cloud data collected by LiDAR; and (b) shows the results after point cloud segmentation. The points marked in red represent ground points while the points marked in others represent segmentation points, and the points that cannot be clustered have been removed; (c) shows the visualization diagram of the edge feature points n***F***_e_ (in blue) and the plane feature points n***F***_p_ (in orange) extracted from ground points or segmentation points according to the value of roughness *c*. The edge feature n***F***_e_ with the largest roughness c, and the non-ground and plane feature n***F***_p_ with the smallest roughness c and the ground are extracted from each row of the subgraph to obtain the edge feature set *F*_e_ and the plane feature set *F*_p_ in all subgraphs, shown as (d).

**Figure 10:**
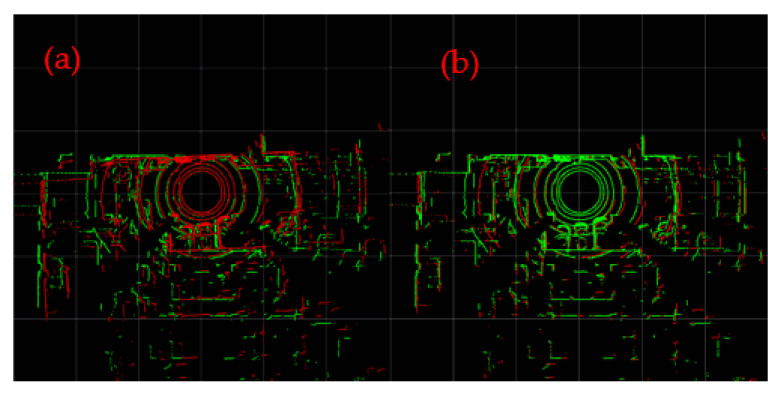
The loopback frames at the entrance of the parking lot and the point cloud of current frame is in green, while the historical loopback frame is in red. (**a**) Detection by the loopback algorithm of LeGo-LOAM itself; (**b**) detection by scan context.

The loopback frames at the entrance of the parking lot are detected respectively by using the traditional LeGO-LOAM and the LeGO-LOAM-SC proposed in this paper; the visualization results are shown in Figure 10. Among them, (a) is the result of loop detection and visual processing by traditional LeGO-LOAM, and (b) is the visual diagram of loop detection by improved LeGO-LOAM-SC. The results show that scan context is used as loopback detection to make the point cloud coincidence degree of the current frame and the detected historical loopback frame higher, indicating that the correction effect of historical pose and map is better and has a better loopback effect.

### 4.1. Mapping Effect

**Figure 11:**
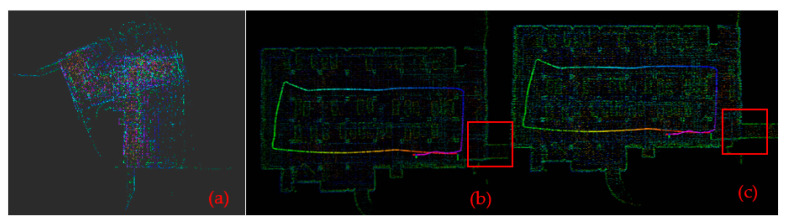
Constructed map for scene #1. (**a**) By LOAM; (**b**) by LeGO-LOAM; (**c**) by LeGO-LOAM-SC.

**Figure 12:**
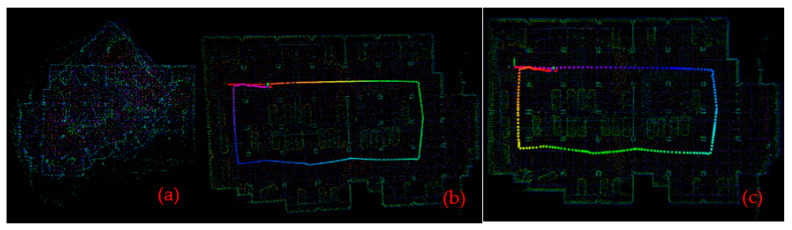
Constructed map for scene #2. (**a**) By LOAM; (**b**) by LeGO-LOAM; (**c**) by LeGO-LOAM-SC.

For the point cloud data for the two scenarios obtained from the experiment, the maps were constructed respectively using three algorithms—LOAM, LeGO-LOAM and LeGO-LOAM-SC—as shown in Figure 11 and Figure 12.

**Figure 13:**
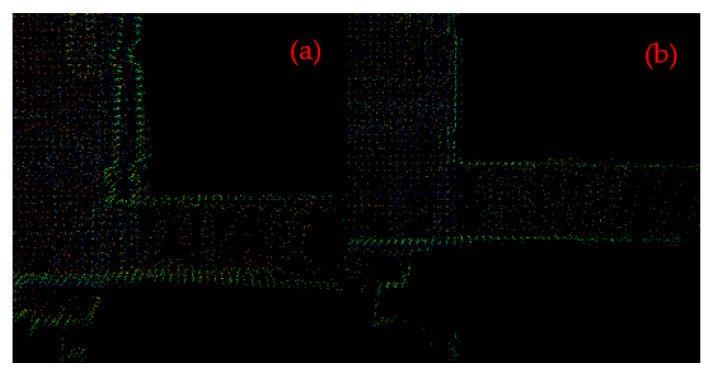
Partial enlargement of the map for scene #1. (**a**) By LeGO-LOAM; (**b**) by LeGO-LOAM-SC.

It can be seen from Figure 11 and Figure 12 that in the mapping test of the two scenarios, the loam algorithm failed to build a complete map; compared with the LeGO-LOAM algorithm, the map constructed by the LeGO-LOAM-SC algorithm is clearer and the mapping effect is better. Figure 13 is the partial enlarged map of the Figure 11 (the part in the red box). It shows that the LeGO-LOAM loopback effect is poor with point cloud map drift and misalignment at the loopback location (Figure 13a), and that the LeGO-LOAM-SC reduces the map drift and coincides at the loop, and the loopback effect is better (Figure 13b).

### 4.2. Trajectory Contrast

**Figure 14:**
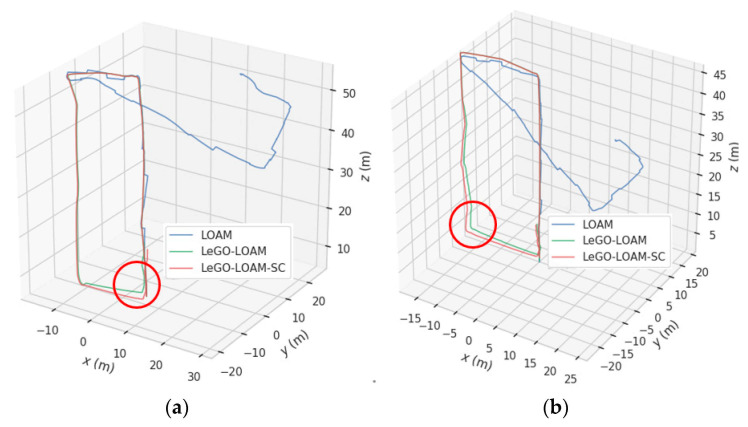
Comparison plots of the LOAM, LeGO-LOAM-SC, and LeGO-LOAM trajectories. (**a**) For scene #1; (**b**) for scene #2.

The motion trajectories were estimated respectively by three algorithms—LOAM, LeGO-LOAM and LeGO-LOAM-SC—and were extracted using an evo tool, as shown in Figure 14. It can be seen from Figure 14 that LOAM cannot trace the trajectory, and that at the place with the large turning angle (circled in red), the LeGO-LOAM trajectory drift is large, while the LeGO-LOAM-SC algorithm trajectory is smoother, and its overall positioning effect is better.

Table 3 shows the relative position estimation error when returning to the starting position. It can be seen from the table that the translation and rotation deviations of the three SLAM algorithms are large, indicating that it is difficult to obtain accurate positioning results only by 3D LiDAR. In comparison, the LeGO-LOAM-SC proposed in this paper can still achieve more accurate positioning and mapping results when only using 3D LiDAR information, and the translation and rotation accuracy of the map is improved by about 5%.

### 4.3. Track Length and Time Consuming

The trajectory length and time-consuming estimated by LeGO-LOAM and LeGO-LOAM-SC were analyzed using the evo tool, as shown in Table 4. LeGO-LOAM-SC has a better real-time performance and reduces time consumption by about 4%.

## 5. Conclusions

(1) An improved SLAM algorithm fusing LeGO-LOAM with scan context, LeGO-LOAM-SC algorithm, is proposed. The data set test and experimental test results show that the improved algorithm has a higher mapping accuracy and a lower time-consumption and resource occupancy.
(2) The KITTI dataset 00 sequence is used to test the mapping and pose estimation performance of LeGO-LOAM and LeGO-LOAM-SC. The results show that LeGO-LOAM-SC improves the drift of the point cloud map, the coincidence degree between motion trajectory estimation and real trajectory is higher, the loop is smoother, the estimated trajectory length is closer to the real trajectory length, and the time consumption is reduced by 2%. The CPU occupancy is reduced by 6%, the maximum error, minimum error and mean square error of ATE are reduced by 49.4%, 79.1% and 55.7% respectively, and the maximum error, minimum error and mean square error of RPE are reduced by 62.9%, 25.0% and 50.3%, respectively.
(3) An experimental test found that the map constructed by LeGO-LOAM-SC algorithm is clearer, the loopback effect is better, the generated estimation trajectory is smoother, the overall positioning is more accurate, the translation and rotation accuracy is improved by about 5%, and the time consumption is reduced by about 4%.

## Figures and Tables

**Table 1:** Track length and the deviation, CPU occupancy and time consumption by the two algorithms.

Algorithm	Path Length (m)	Track Length Deviation (m) (Actual Path Length is 3724.187 m)	The CPU Occupancy Rate (%)	Time Consumption (s)
LeGO-LOAM	3730.692	6.505	65	570.167
LeGO-LOAM-SC	3727.583	3.396	61	558.564

**Table 2:** Comparison of absolute trajectory error and relative position error.

Algorithm	Evaluating Indicator	Maximum Error	Minimum Error	Mean Square Error
LeGO-LOAM	ATE	11.177 m	0.885 m	4.976 m
	RPE	6.173	0.004	0.159
LeGO-LOAM-SC	ATE	5.652 m	0.185 m	2.206 m
	RPE	2.289	0.003	0.079

**Table 3:** The estimation error when returning to the starting position.

Scene	#1			#2		
Algorithm	LOAM	LeGO-LOAM	LeGO-LOAM-SC	LOAM	LeGO-LOAM	LeGO-LOAM-SC
Translation X (m)	22.68	−0.86	0.40	20.36	−1.24	−0.98
Translation Y (m)	3.57	0.14	0.10	−2.83	0.56	0.09
Translation Z (m)	59.84	13.86	12.23	38.32	8.92	8.59
Total translation (m)	64.09	13.89	12.24	43.49	9.02	8.65
Pitch angle (deg)	−69.09	−2.46	0.93	−75.87	−6.36	−5.18
Drift angle (deg)	8.01	0.74	−6.26	13.02	−6.47	2.51
Roll angle (deg)	2.86	6.27	−0.58	−26.83	4.43	1.26
Total rotation (deg)	70.42	6.78	6.39	81.52	10.06	5.89

**Table 4:** Track length and time-consuming.

Scene	Algorithm	Path Length (M)	Time Consuming (S)
#1	LeGO-LOAM	186.954	220.364
	LeGO-LOAM-SC	179.286	211.864
#2	LeGO-LOAM	192.258	234.950
	LeGO-LOAM-SC	198.360	225.268

## References

[1] Wang G., Zhang D. (2018). Innovation practice and development prospect of intelligent fully mechanized coal mining Technology. J. China Univ. Min. Technol..

[2] Forster C., Pizzoli M., Scaramuzza D. SVO: Fast semi-direct monocular visual odometry. Proceedings of the 2014 IEEE International Conference on Robotics and Automation (ICRA 2014).

[3] Engel J., Koltun V., Cremers D. (2018). Direct sparse odometry. IEEE Trans. Pattern Anal. Mach. Intell..

[4] Qin T., Li P., Shen S. (2018). Vins-mono: A robust and versatile monocular visual-inertial state estimator. IEEE Trans. Robot..

[5] Campos C., Elvira R., Rodríguez J.J.G., Montiel J.M., Tardós J.D. (2021). ORB-SLAM3: An Accurate Open-Source Library for Visual, Visual–Inertial, and Multimap SLAM. IEEE Trans. Robot..

[6] Rusu R., Bradski G., Thibaux R., Hsu J. Fast 3d recognition and pose using the viewpoint feature histogram. Proceedings of the 2010 IEEE/RSJ International Conference on Intelligent Robots and Systems.

[7] Fernandes D., Afonso T., Girão P., Gonzalez D., Silva A., Névoa R., Novais P., Monteiro J., Melo-Pinto P. (2021). Real-Time 3D Object Detection and SLAM Fusion in a Low-Cost LiDAR Test Vehicle Setup. Sensors.

[8] Zhao S., Fang Z., Li H., Scherer S. A robust laser-inertial odometry and mapping method for large-scale highway environments. Proceedings of the 2019 IEEE/RSJ International Conference on Intelligent Robots and Systems (IROS).

[9] Shan T., Englot B., Meyers D., Wang W., Ratti C., Rus D. Lio-sam: Tightly-coupled lidar inertial odometry via smoothing and mapping. Proceedings of the 2020 IEEE/RSJ International Conference on Intelligent Robots and Systems (IROS).

[10] Liu Z., Zhang F. (2021). Balm: Bundle adjustment for lidar mapping. IEEE Robot. Autom. Lett..

[11] Zhou Z., Cao J., Di S. (2021). Overview of 3D Lidar Slam Algorithms. Chin. J. Sci. Instrum..

[12] Seetharaman G., Lakhotia A., Blasch E.P. (2006). Unmaned Vehicle come of age: The DARPA grand challenge. Computer.

[13] Meng D., Tian B., Cai F., Gao Y., Chen L. (2021). Road slope real-time detection for unmanned truck in surface mine. Acta Geod. Cartogr. Sin..

[14] Rahman M.F.F., Fan S., Zhang Y., Chen L. (2021). A Comparative study and application of unmanned agricultural vehicle system in agriculture. Agriculture.

[15] Hu Y., Wang X., Hu J., Gong J., Wang K., Li G., Mei C. (2021). An overview on unmanned vehicle technology in off-road environment. Trans. Beijing Inst. Technol..

[16] Huber D.F., Vandapel N. (2006). Automatic three-dimensional underground mine mapping. Int. J. Robot. Res..

[17] Ren Z., Wang L., Bi L. (2019). Robust GICP-based 3D LiDAR SLAM for underground mining environment. Sensors.

[18] Segal A., Haehnel D., Thrun S. Generalized-icp. Proceedings of the Robotics: Science and Systems V, University of Washington.

[19] Zhang J., Singh S. LOAM: Lidar Odometry and Mapping in Real-time. Proceedings of the Robotics: Science and Systems Conference (RSS).

[20] Zhang J., Singh S. (2017). Low-drift and real-time lidar odometry and mapping. Auton. Robot..

[21] Shan T., Englot B. Lego-loam: Lightweight and ground-optimized lidar odometry and mapping on variable terrain. Proceedings of the 2018 IEEE/RSJ International Conference on Intelligent Robots and Systems (IROS).

[22] Koide K., Miura J., Menegatti E. (2019). A portable three-dimensional LIDAR-based system for long-term and wide-area people behavior measurement. Int. J. Adv. Robot. Syst..

[23] Biber P., Straßer W. The normal distributions transform: A new approach to laser scan matching. Proceedings of the 2003 IEEE/RSJ International Conference on Intelligent Robots and Systems (IROS 2003) (Cat. No. 03CH37453).

[24] Deschaud J.E. IMLS-SLAM: Scan-to-model matching based on 3D data. Proceedings of the 2018 IEEE International Conference on Robotics and Automation (ICRA).

[25] Behley J., Stachniss C. Efficient Surfel-Based SLAM using 3D Laser Range Data in Urban Environments. Proceedings of the Robotics: Science and Systems.

[26] Liu X., Zhang L., Qin S., Tian D., Ouyang S., Chen C. (2019). Optimized LOAM Using Ground Plane Constraints and SegMatch-Based Loop Detection. Sensors.

[27] Rusu R.B., Blodow N., Beetz M. Fast point feature histograms (FPFH) for 3D registration. Proceedings of the 2009 IEEE International Conference on Robotics and Automation.

[28] Bosse M., Zlot R. Place recognition using keypoint voting in large 3D lidar datasets. Proceedings of the 2013 IEEE International Conference on Robotics and Automation.

[29] Rizzini D.L. Place recognition of 3D landmarks based on geometric relations. Proceedings of the 2017 IEEE/RSJ International Conference on Intelligent Robots and Systems (IROS).

[30] Kim G., Kim A. Scan context: Egocentric spatial descriptor for place recognition within 3d point cloud map. Proceedings of the 2018 IEEE/RSJ International Conference on Intelligent Robots and Systems (IROS).

[31] Evo: Python Package for the Evaluation of Odometry and SLAM. https://github.com/MichaelGrupp/evo.

[32] Huang W., Li Y., Hu F. Real-Time 6-DOF Monocular Visual SLAM based on ORB-SLAM2. Proceedings of the 2019 Chinese Control and Decision Conference (CCDC).

